# Point-of-care testing as a catalyst for malaria elimination in Africa

**DOI:** 10.4102/ajlm.v15i1.3449

**Published:** 2026-10-14

**Authors:** Glenda M. Davison, Akin Abayomi, Rajiv T. Erasmus

**Affiliations:** 1 SAMRC/CPUT Cardiometabolic Health Research Unit, Faculty of Health and Wellness, Cape Peninsula University of Technology, Cape Town, South Africa; 2 Department of Biomedical Sciences, Faculty of Health and Wellness Sciences, Cape Peninsula University of Technology, Cape Town, South Africa; 3 Division of Haematology, Faculty of Health, University of Cape Town, Cape Town, South Africa; 4 Lagos State Ministry of Health, Lagos State Government, Lagos, Nigeria; 5 Division of Haematology, Faculty of Medicine and Health Sciences, Stellenbosch University, Cape Town, South Africa; 6 Division of Chemical Pathology, Faculty of Medicine and Health Sciences, Stellenbosch University, Cape Town, South Africa

The World Health Organization (WHO) estimates that 282 million malaria cases are reported annually with 95% occurring in Africa and young children accounting for 75% of deaths.^1^ In 2015, the WHO implemented a strategy to reduce malaria by 90%^2^; however, despite efforts, delayed diagnoses, missed cases and antimicrobial resistance have led to continued transmission. This has been compounded by genetic adaptation of parasites, climate change, weak health systems and poor disease surveillance.^3^

In sub-Saharan countries, fevers are treated presumptively as malaria owing to historically high prevalences and childhood mortality. Treating presumed malaria without diagnostic confirmation results in mass drug administration and high artemisinin-based combination therapy (ACT) residues in humans and the environment. Under these conditions, resistant parasites multiply and are transmitted, contributing to the spread of resistant variants.^4^

Microscopy remains the gold standard for diagnosis but use in under-resourced settings is limited by the need for reliable electricity, infrastructure and skilled personnel.^5^ Alternatively, point-of-care tests (POCT) can transform malaria management by providing a simple test that is rapid and affordable. Antigen rapid diagnostic tests (RDTs) are widely used as they are cost effective, require finger-prick blood, are not reliant on electricity and can provide a result within 10–15 min.^5^

Most RDTs target the *Plasmodium falciparum*-specific histidine-rich protein 2 (HRP2) or parasite lactate dehydrogenase (pLHD), making them effective in sub-Saharan Africa where *P. falciparum* predominates.^5^ However, accuracy is compromised by supply chain logistics and the emergence of *P. falciparum* strains with hrp2/hrp3 gene deletions. Conventional RDTs have lower sensitivity with asymptomatic patients often being missed, but newer, highly sensitive RDTs have improved detection allowing low parasitaemia to be identified. Rapid diagnostic tests cannot measure parasite density and HRP2 antigen persistence after treatment can lead to false positive results.^6^ Loop-mediated isothermal amplification (LAMP) and polymerase chain reaction (PCR) have overcome these limitations but are costly and dependent on electricity, restricting their use in low-resourced settings.^5^

Despite this, RDTs have the potential to make a significant impact on achieving the WHO goal of reducing the burden of malaria.^2^ RDTs can be used for tracking and identifying hotspots, allowing healthcare services to allocate resources and control outbreaks.^7^ In this context; RDTs are not only a clinical tool but can also be utilised in surveillance and play a role in the elimination of malaria.

One practical example is the IMPACT project, also known as Immunization Plus, with a sub-activity project known as The Pathway to Malaria Pre-Elimination and Digitization Project.^8^ The objective was to define a simple and reliable diagnostic tool and deploy it to define the true malaria burden in the community. Lagos is a densely populated riverine region of Nigeria with a population of 30 million. Malaria had a reported prevalence of 15% in 2010,^9^ but after effective malaria programmes, the State has seen a reduction in prevalence to 3% in 2025. Despite this, medical facilities were still diagnosing elevated levels of malaria, a clinical pattern of febrile management inherited without understanding the changing physical and demographic narratives.

Once the accuracy of the WHO pre-qualified RDTs was established, they were deployed in a larger study to define the current burden of malaria and its role in febrile illness. The results revealed that Malaria was only responsible for 5% of all fevers. Lagos Ministry of Health, then deployed a ‘test before treatment’ strategy across all government facilities, which resulted in a dramatic decline of patients diagnosed and treated for malaria. A digital tracking system allowed for the identification of clusters and targeted approaches. In addition, the Ministry of Health invested in malaria control measures, strengthened public health systems, and partnered with the private sector.^10^

Recent surveys published by the WHO, have suggested that Lagos had a prevalence rate approaching 2% with movement towards pre-elimination.^11^ The experience of Lagos matters because it is a catalyst to rethinking Malaria across the country and the sub-region. It illustrates how adopting RDTs at scale combined with surveillance and a well-coordinated public health policy can change the management of febrile illnesses. It revealed the true burden of active disease and encouraged evidence-based policy to accelerate control and elimination.

Despite the success of Lagos, there are still challenges hindering a large-scale ‘test, treat and track’ programme being implemented throughout Africa. Barriers include supply chain failures, limited funding, poor quality assurance, lack of training and insufficient surveillance. For malaria programmes to succeed, training, assessment and monitoring must form part of quality assurance. Training should include sample collection, storage, biosafety, quality control and the correct response to both positive and negative results.

When point-of-care testing (POCT) is integrated into prevention strategies with governmental support, it can strengthen a ‘test, treat and track’ approach and ensure that reliable diagnostics are brought to communities most affected by the disease. This approach could be a powerful tool in the continent’s journey towards malaria elimination.
